# Virtual home visits training regarding personal safety for postgraduate trainees in general practice – learning gain and feelings of anxiety with video and 360° VR applications

**DOI:** 10.1186/s12909-025-07995-x

**Published:** 2025-10-02

**Authors:** Luisa Maichle, Jost Steinhäuser, Kristina Flägel

**Affiliations:** Institute of Family Medicine, University Hospital Schleswig-Holstein, Campus Luebeck, Ratzeburger Allee 160, Lübeck, 23538 Germany

**Keywords:** Home visits, Digital learning, Postgraduate training, General practice, Virtual reality

## Abstract

**Background:**

Performing home visits needs to be trained with regards to the general practitioners’ safety in unknown environment. Since real home visits cannot be standardized, we aimed for the implementation of virtual home visits into postgraduate training.

**Methods:**

We conducted a controlled pilot study with postgraduate trainees in general practice taking part in 90-minute seminars on personal safety aspects during home visits. They performed a virtual home visit in form of a video or 360° virtual reality (VR). A pre-post evaluation was used to measure the learning gain via self-assessment and state anxiety. This pilot study was not registered and therefore, has no trial number.

**Results:**

Virtual home visits resulted in an increase in confidence to carry out home visits independently (z-value = 28.229, *p* < 0.001 in the video group (*n* = 92) and means 3.1 (t0), 3.4 (t1) and 4.1 (t2) in the VR group (*n* = 11)) and changes in being scared of performing home visits on their own in the VR group (means 2.1 (t0), 2.3 (t1) and 3.0 (t2)).

**Conclusions:**

Virtual home visits are able to prepare trainees for performing home visits in seminar group size. The use of VR gave no indication of an increased learning gain over video, but appeared more challenging in the implementation, e.g. time-consuming introduction into hard- and software.

**Supplementary Information:**

The online version contains supplementary material available at 10.1186/s12909-025-07995-x.

## Background

If medically indicated, physicians in Germany are under the obligation to visit their patients in their home [1]. Home visits have a high priority for patients who suffer from health deteriorations or chronic conditions that reduce their mobility [2]. With today’s medical technology available for outpatient home visits, costly transport to hospital including the risk of acquiring multi-resistant pathogens can be significantly reduced [3]. Besides that, home visits can strengthen the relationship between physicians and patients, and also their caregivers. Hence, treatment efforts turn out to be more suitable, feasible and helpful [4]. Besides all these positive aspects, 39% of German general practitioners (GPs) stated to have experienced aggression during home visits, 66% of female and 34% of male GPs felt little or no sense of security during home visits while on emergency duty [5]. Feelings of safety and security during home visits might be easily compromised by animals, unfamiliar surroundings that are potentially dangerous, or patients or caregivers reacting aggressively due to medical reasons, psychiatric crises, or drug use [6]. Exposure to violence, particularly during home visits, was highlighted in studies in China and Australia [7–9]. This is especially a major issue for primary healthcare. E.g. GPs in the United Kingdom (UK) or Turkey are faced with occupational violence and aggression; especially female and young GPs are at increased risk [10–12]. This is especially concerning in the context of the growing proportion of female doctors, misleadingly also named “feminization” of medicine [13–16]. This is particularly evident in the GP specialty: data from Germany shows that GP has the highest proportion of women among medical specialties [17].

A systematic review that included studies from all over the world found a prevalence of workplace violence against general practitioners of 63% non-physical violence and 9% physical violence with increasing physical violence with study period [18]. These findings raise concern as they highlight a lasting detrimental effect of workplace violence on general practitioners’ clinical performance and psychological state [19]. The WHO defines “violence and harassment” as “incidents involving work-related abuse, threats or assaults among health workers including physical, sexual, verbal and psychological abuse and workplace harassment” [20]. The WHO recommends to monitor violence trends and the effectiveness of preventive measures [20]. For example, the Royal Australian College of General Practitioners published a guide for the prevention and management of patient-initiated violence in 2015 with the aim to keep general practice a safe place [21]. Workplace violence prevention training is not generally introduced into German medical school curricula or medical postgraduate training regulations [22, 23], wherefore there might be an even larger gap in preparation, although 50 home visits need to be documented during postgraduate training [23].

Regarding the educational potential, the inclusion of home care education into the medical school curriculum has shown, amongst others, to reveal misconceptions about treatments and to enable a return to the roots of medicine [24]. Likewise, delivering structured home visits in undergraduate education and postgraduate training is complicated by the fact that it depends on the general practitioner who performs the home visits with the student and postgraduate trainee, respectively. Real home visits in seminar size groups of trainees do not seem feasible with regards to patient’s privacy and scheduling. Thus, first experiences with video home visits for falls assessment were introduced in medical education and found to be feasible [25].

Technology at the workplace is able to support workplace learning and doctors’ decision-making, and hence, supports postgraduate training [26]. Digital learning has been shown to promote motivation of students in educational settings and increases the learning gain [27, 28], it can also help improve skills and knowledge in academic settings [29]. Virtual reality (VR) is one method of digital enhancement. It is being used more and more in medical education, and it is well received by medical students [25]. VR technology already supports successfully occupational therapists in the home modification process of patients in need [30].

As previously mentioned, postgraduate GP training in Germany has a gap with regard to home visits and, more specifically, personal safety. Our training aims to address the increased potential risks associated with these visits. Systematic reviews have confirmed that educational meetings can lead to the implementation of content in practice, improving professional practice and even resulting in improved patient health [31, 32]. The most effective design appears to be a combination of interactive and instructional elements, a feature that we have incorporated into our seminar structure [32]. Considering that exposure to potentially unpleasant or dangerous simulations in medical training can lead to stress or anxiety hindering the absorption of learning content [33, 34] immersive technology like videos and VR needs to be cautiously included into the training of personal safety aspects during home visits.

The aim of this pilot study was to evaluate the learning gain and the feeling of anxiety of postgraduate trainees in general practice during and after a virtual home visit seminar on personal safety both with video and 360° VR applications.

## Methods

### Seminar design

**Fig. 1:**
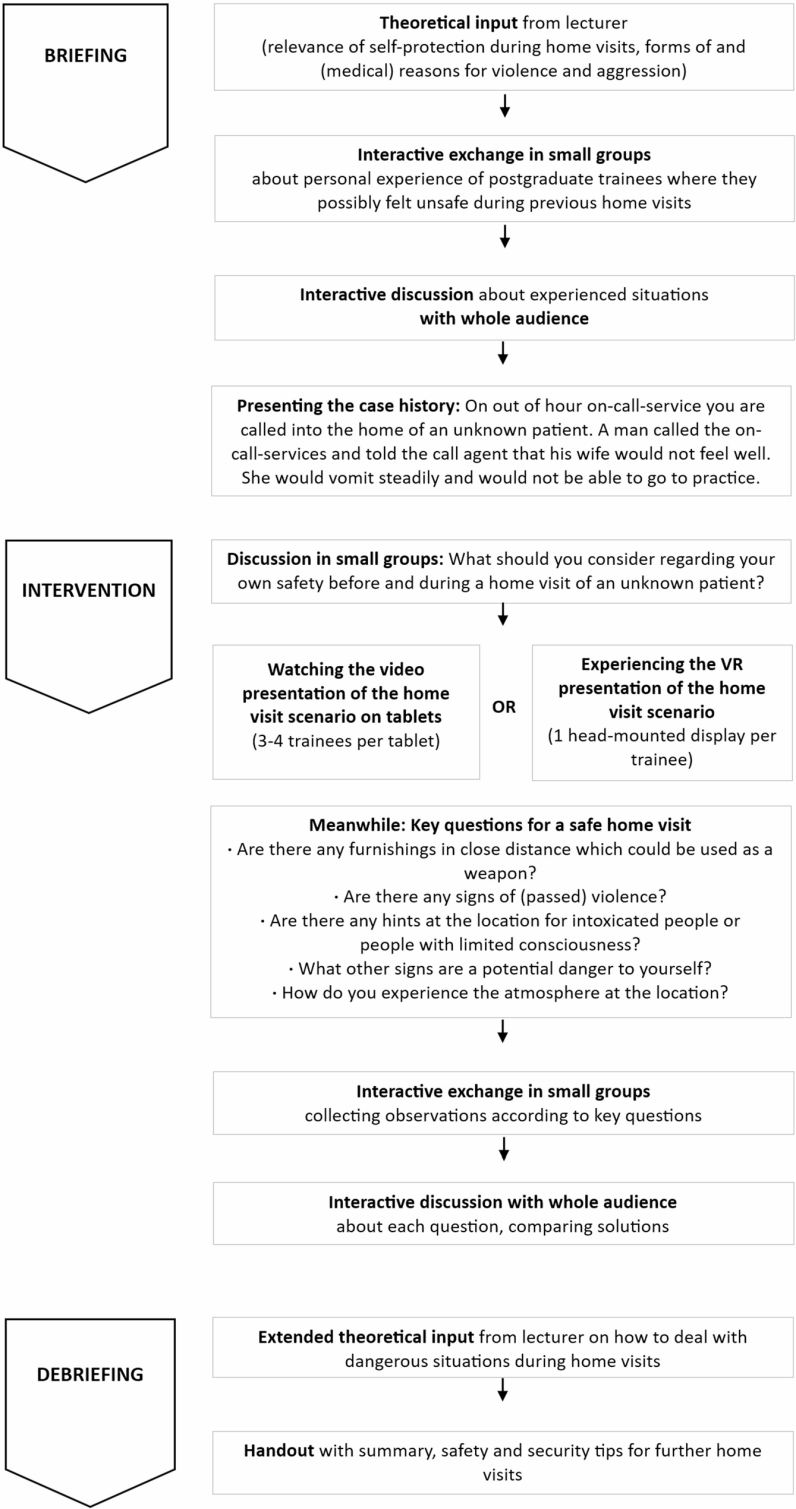
Course of action of the seminar on personal safety during home visits

The learning objective of the 90-minute-seminar on personal safety during home visits was to recognise signs in the patient’s home that indicate possible violence or safety issues and to react properly to those clues. Figure 1 shows more information on the course of action of the seminar, which included briefing, intervention and debriefing as suggested for simulation training [35]. The procedure and content of the seminar was the same for the participants of both groups, only the intervention differed in the choice of technique.

To get an impression of whether the use of VR would have a significant advantage over videos, we ran seminars with either video or VR presentation.

### Creation of video and VR scenarios

A house in the City of Luebeck, Germany, was realistic decorated based on experiences (home visits and emergency medicine) of the authors amongst others with bottles of alcohol, different drugs, weapons, a furnace, and dog accessories. After the setup a video was taken by going through the premises with a Canon EOS 200D.

For the VR scenario, multiple pictures with a 360° Insta One X2 were taken stepwise. The Institute of Robotics at the University Luebeck assembled those pictures into a VR scenario, in which the participant can take steps through the premises by pulling the trigger of the Meta Quest 2 controller using head-mounted displays (HMD).

### Recruitment

The seminars on personal safety during home visits were integrated into the educational meetings of the Competence Centres in Postgraduate Training General Medicine Schleswig-Holstein and Mecklenburg-Vorpommern in November and December 2022 and May 2023, respectively. These educational meetings address various essential aspects of the GP profession in seminars (36, 37). GP postgraduate trainees – licensed physicians in their five-year postgraduate training to become a general practitioner – took part in those seminars. All for the home visit seminar signed up postgraduate trainees were then invited to voluntarily take part in the pilot study by filling out the questionnaires for evaluation purposes. Because of organizational reasons (preformed groups of trainees that signed up for the seminars), no randomization was possible. All participants in the federal state Schleswig-Holstein received the video home visit, all participants in Mecklenburg-Vorpommern received the 360° VR home visit.

### Evaluation concept

Evaluation concept was built as pre-post-evaluation with a questionnaire right at the beginning of the seminar (t0), right after the seminar (t1) and two months later (t2). The first two were handed out to the study participants as paper questionnaires, t2 was prepared as online questionnaire (SurveyMonkey). The link for t2 was sent via mail to participating trainees with an incentive of a 20-Euro voucher. A reminder was sent two weeks after the initial mail. Data was collected by using a pseudonym.

This questionnaire was developed for this study. It included a 9-item comparative self-assessment (CSA) regarding competencies relevant for performing home visits considering the seminar’s learning objectives to inform about the learning gain induced by the seminars. In order to assess the emotional response to the virtual home visit the German 10-item short version representing the state anxiety scale of the State-Trait-Anxiety Inventory (STAI) was added (38–40).

Sociodemographic items included gender, age, year of postgraduate training in general practice, previous medical training, participant’s experience with home visits, and experience with VR. A free text for comments was made available to the participants. The questionnaire is available as supplementary file in an English language version [see Additional file 1].

Next to quantitative data the lecturer’s observations of critical aspects in virtual home visit seminar implementation were documented.

### Statistical analysis

Statistical analysis was performed with IBM SPSS Statistics for Windows, Version 28.0 (Armonk, NY: IBM Corp.). Next to descriptive statistics (absolute numbers and frequencies, means and standard deviations) for the video and VR group inductive statistics were used for the video group. Since data were not normally distributed, Friedman test was used for calculating differences in the self-assessment between t0, t1 and t2. Since participation was, like anticipated, lower in t2 in comparison to t0 and t1, only complete data sets from participants (responses in t0, t1 and t2) were included in the analyses. Dunn-Bonferroni tests were used as post-hoc tests to reveal significant differences between single measure points. Wilcoxon signed-rank test was used for items that were measured twice, e.g. items from STAI in t0 and t1. McNemar test was used to determine if there were differences between the number of postgraduate trainees who performed home visits on their own from t0 to t2. Effect sizes were calculated and interpreted according to Cohen (1992). The significance level was set at 0.05.

## Results

In total, 162 postgraduate trainees attended ten seminars with virtual home visits: 142 with video, 20 with VR. Since participation in t2 was lower than in t0 and t1, complete data sets from 92 trainees in the video group and 11 trainees in the VR group were included in the analyses. Remarkably, two postgraduate trainees dropped out of the study because they withdrew their participation from the home visit in virtual reality. Prior motion sickness symptoms with VR applications were the reason not to try it during the seminars. In comparison, there were no dropouts in the virtual home visit via video. The participants in the video group had more previous medical training than participants in the VR group (50% vs. 18%) and were more experienced in performing home visits on their own (85% vs. 55%). Participants of the video group were on average five years younger. Participants of both groups were on average in their fourth year of postgraduate training. Table 1 shows more details.

**Table 1:** Sociodemographic characteristics of participants in the home visit seminar with virtual home visit via video or via VR

	Video group (*n* = 92)	Virtual reality group (*n* = 11)
	*N* (%)	*N* (%)
**Gender**		
Male	21 (23)	3 (27)
Female	70 (76)	8 (73)
Diverse	-	-
Missing	1 (1)	-
**Year of Birth**		
1950–1969	3 (3)	-
1970–1979	16 (17)	-
1980–1989	49 (53)	5 (46)
1990–1999	23 (25)	6 (54)
Missing	1 (1)	-
**Year of postgraduate training in general practice**		
1	7 (8)	-
2	8 (9)	2 (18)
3	17 (19)	4 (36)
4	26 (28)	1 (9)
5	34 (37)	4 (36)
**Previous medical training**		
None	46 (50)	9 (82)
(Paediatric) Nursing	21 (23)	-
Emergency medical services	7 (8)	-
Therapeutic professions (e.g. physiotherapy)	7 (8)	-
Specialist in another medical discipline	7 (8)	-
Other medical training	4 (4)	2 (18)
**Previous home visit experiences**		
Already performed on my own	78 (85)	6 (55)
Home visits experienced as a companion	55 (60)	10 (91)
Dealt with home visits theoretically	16 (17)	5 (46)
Never got to know home visits	3 (3)	-
**Previous VR experiences**		
Never used	81 (88)	8 (73)
Sometimes used	10 (11)	3 (27)
Regularly using	1 (1)	-
	**Mean (SD)**	**Mean (SD)**
**Year of birth**	1984 (7,0)	1989 (2,3)
**Year of postgraduate training in general practice**	4 (1,2)	4 (1,2)
**Previous VR experiences**	0,2 (0,72)	0,3 (0,47)

The added percentage values may deviate from 100% due to rounding

### Results of the virtual home visit via video

In the CSA, postgraduate trainees described themselves as more confident to recognise and make recommendations on situations jeopardizing the safety of their patients in their home, to identify and respond adequately to dangerous situations for themselves during a home visit, to gather accumulated knowledge of the patient, the patient’s values and the patient’s family history and to perform home visits (*p* < 0.001).

Post-hoc testing showed that identifying and responding adequately to dangerous situations for themselves differed significantly between t0 and t1 as well as t0 and t2 (*p* < 0.001). The assessment of recognising situations jeopardizing the safety of their patients in their home differed significantly between t0 and t1 (*p* = 0.002) and t0 and t2 (*p* = 0.004). Gathering accumulated knowledge of the patient, the patient’s values and the patient’s family history and performing home visits differed between t1 and t2 (*p* = 0.005 and *p* < 0.001, respectively) and t0 and t2 (*p* < 0.001 and *p* = 0.002, respectively). Making recommendations on situations jeopardizing the safety of their patients in their home differed significantly between t0 and t2 (*p* = 0.004). Effect sizes were small ranging between *r* = 0.048 to *r* = 0.075.

Postgraduate trainees felt medically and socially more prepared in t2 (*p* < 0.001). Effect sizes were large with *r* = 0.586 and *r* = 0.402 respectively.

After two months participants felt significantly more prepared for home visiting, medically and socially. Still, with means ranging from 3.3 to 4.1 not all postgraduate trainees felt confident or well prepared. The seminar did not change their self-assessment in being scared of performing home visits on their own.

In STAI, feeling rested and pleasant decreased after the seminar (*p* = 0.031 and *p* = 0.048 respectively). Effect sizes were small (*r* = 0.225 and *r* = 0.206). All other items did not show statistically significant changes. Table 2 displays more information.

**Table 2:** Statistical analysis of the virtual home visit via video

	t0 Mean (SD)	t1 Mean (SD)	t2 Mean (SD)	N*	test statistic	*p* value	effect size
**CSA**	*0 = not at all − 5 = very much*						
I feel confident to recognise situations jeopardizing the safety of my patients in their home.	3.3 (1.11)	3.8 (0.74)	3.8 (0.69)	92	χ^2^(2) = 26.380	**< 0.001**	r_t0_t1_ = 0.052 r_t0_t2_ = 0.050
I feel confident to be able to make recommendations on situations jeopardizing the safety of my patients in their home.	3.2 (1.02)	3.5 (0.85)	3.6 (0.79)	92	χ^2^(2) = 18.768	**< 0.001**	r_t0_t2_ = 0.047
I feel confident to identify dangerous situations for myself during a home visit.	3.1 (1.21)	3.9 (0.76)	3.9 (0.71)	91	χ^2^(2) = 45.246	**< 0.001**	r_t0_t1_ = 0.069 r_t0_t2_ = 0.067
I feel confident to be able to respond adequately to dangerous situations for myself during a home visit.	2.5 (1.10)	3.3 (1.00)	3.3 (0.97)	92	χ^2^(2) = 45.206	**< 0.001**	r_t0_t1_ = 0.075 r_t0_t2_ = 0.074
I feel confident to gather accumulated knowledge of the patient, the patient’s values and the patient’s family history.	3.5 (0.91)	3.7 (0.81)	4.1 (0.68)	92	χ^2^(2) = 39.654	**< 0.001**	r_t0_t2_ = 0.063 r_t1_t2_ = 0.048
I feel medically well prepared for performing home visits.	2.8 (1.11)	-	3.5 (1.03)	92	Z = −5.622	**< 0.001**	*r* = 0.586
I feel socially well prepared for home visiting.	3.7 (0.94)	-	4.0 (0.83)	92	Z = −3.855	**< 0.001**	*r* = 0.402
I feel confident to perform home visits.	3.6 (0.94)	3.5 (1.14)	4.0 (0.95)	91	χ^2^(2) = 28.229	**< 0.001**	r_t0_t2_ = 0.052 r_t1_t2_ = 0.062
I am scared of performing home visits on my own.	1.6 (1.21)	1.7 (1.17)	1.7 (1.31)	92	χ^2^(2) = 2.846	0.241	
**STAI**	***0 = not at all – 6 = very much so***						
I feel calm.	4.5 (1.07)	4.4 (1.33)	-	91	Z = -0.958	0.338	
I am tense.	1.7 (1.42)	1.6 (1.49)	-	91	Z = -1.075	0.282	
I feel upset.	1.5 (1.24)	1.4 (1.43)	-	91	Z = −1.108	0.268	
I feel rested.	3.0 (1.77)	2.8 (1.65)	-	92	Z = −2.160	**0.031**	*r* = 0.225
I feel anxious.	1.2 (1.51)	1.4 (1.44)	-	92	Z = −1.470	0.141	
I feel self-confident.	3.9 (1.14)	4.0 (1.26)	-	92	Z = −0.289	0.773	
I feel nervous.	1.3 (1.38)	1.1 (1.24)	-	92	Z = −1.417	0.156	
I feel “high-strung.”	1.0 (1.09)	0.9 (1.11)	-	92	Z = −0.598	0.550	
I am worried.	1.4 (1.33)	1.4 (1.40)	-	92	Z = −0.578	0.563	
I feel pleasant.	3.5 (1.24)	3.2 (1.29)	-	92	Z = −1.975	**0.048**	*r* = 0.206

*N: Number of responding participants

The indication of performing home visits on their own did not change significantly from t0 to t2 (*p* = 0.125).

### Results of the virtual home visit via VR

The VR group starts with lower means in the CSA than the video group except for being more scared of performing home visits on their own. All means in the CSA increase in t1 and again from t1 to t2, including being scared of performing home visits. The results in STAI remain mostly stable with clear reductions in feeling upset (mean 2.6 in t0 to 1.3 in t1) and feeling nervous (mean 1.8 in t0 to 1.0 in t1). More details are displayed in Table 3.

**Table 3:** Descriptive statistics of the virtual home visit via virtual reality

	t0 Mean (SD)	t1 Mean (SD)	t2 Mean (SD)
**CSA**	***0 = not at all − 5 = very much***		
I feel confident to recognise situations jeopardizing the safety of my patients in their home.	2.6 (1.21)	3.4 (0.92)	3.7 (0.79)
I feel confident to be able to make recommendations on situations jeopardizing the safety of my patients in their home.	2.4 (0.92)	3.0 (1.10)	3.6 (1.04)
I feel confident to identify dangerous situations for myself during a home visit.	2.6 (0.82)	3.4 (1.21)	3.7 (0.79)
I feel confident to be able to respond adequately to dangerous situations for myself during a home visit.	2.1 (0.83)	2.6 (1.12)	3.6 (0.67)
I feel confident to gather accumulated knowledge of the patient, the patient’s values and the patient’s family history	2.8 (0.87)	3.5 (1.13)	4.5 (0.52)
I feel medically well prepared for performing home visits.	2.3 (0.91)	-	3.4 (1.12)
I feel socially well prepared for home visiting.	3.4 (1.21)	-	4.1 (0.70)
I feel confident to perform home visits.	3.1 (1.30)	3.4 (0.92)	4.1 (0.83)
I am scared of performing home visits on my own.	2.1 (1.38)	2.3 (1.10)	3.0 (1.41)
**STAI**	***0 = not at all – 6 = very much so***		
I feel calm.	3.8 (0.98)	3.9 (1.45)	-
I am tense.	2.2 (1.25)	2.0 (1.48)	-
I feel upset.	2.6 (1.43)	1.3 (0.91)	-
I feel rested.	2.9 (1.30)	3.0 (0.89)	-
I feel anxious.	1.6 (1.29)	1.6 (1.37)	-
I feel self-confident.	3.3 (1.27)	3.8 (1.25)	-
I feel nervous.	1.8 (1.40)	1.0 (1.18)	-
I feel “high-strung.”.	1.1 (1.14)	1.0 (0.78)	-
I am worried.	1.4 (1.43)	1.6 (1.13)	-
I feel pleasant.	3.3 (0.65)	3.6 (1.21)	-

In t2, 22% of participants in the video group (*n* = 20) agreed absolutely that they would like to use VR for continuing medical education whereas 54% in the VR group (*n* = 6) did so.

### Lecturer’s observations of critical aspects in virtual home visit seminar implementation

Compared to the video group, introducing the HMD and the controllers as well as the functions of the VR software were more time-consuming. It took about 15 min extra. Moreover, transport of hardware (15 Meta Quest 2 versus 5 iPads) was more challenging. Even packed in cases, HMDs and controllers for every participant took up a lot more space than iPads. This made transport and use in different locations especially challenging.

## Discussion

In this pilot study, the aim was to implement and evaluate two different forms of virtual home visits (video and 360° VR) in the postgraduate training of GPs considering personal safety during home visits.

Participants stated that they felt more confident to recognise and respond to dangerous situations for themselves after the virtual home visits via video and via VR. The lasting effect in t2 may be due to the impact of our virtual home visits. It is known and has been successfully replicated that the learning success after 31 days is only about 20% (41, 42), which means that the results of our data after two months may be explained by postgraduate trainees dealing with home visits after the seminar and using the learning content that was given on a regular basis. It might be concluded that this seminar was appropriate for the target group in the sense that they were able to use the learning content right away.

Another aim of the evaluation was to find out whether direct confrontation with personal safety aspects during home visits triggers negative emotions such as fears or anxiety, because the up-close experience by digital media such as videos or even VR may intensify the effects [33]. It is also known that simulations in medical training in general can lead to increased stress and anxiety among participants [43]. It might be a counterproductive result of such a seminar for prospective general practitioners regarding future home visits, but also for keeping the teaching success of a seminar high, as learning is essentially dependent on the mind, condition, and motivation of the participants [44]. In the video group, there was no significant increase or decrease in being scared of performing home visits on their own, neither immediately after the seminar nor two months later. This leads us to believe that teaching personal safety during home visits using videos does not lead to increased fear of such situations. In the VR group there is a slight increase in the self-assessment of being scared from t0 to t1 and an even bigger increase from t1 to t2. Whether this is an artefact of self-assessment or the product of the VR application needs to be assessed in further studies with more participants that undergo the VR seminar. A time-delayed learning gain using VR has not been reported so far.

Some significant results in the STAI in the video group were an observed decrease in feeling rested and pleasant. These changes could, after all, be indications that dealing with the unpleasant topic revealing potential dangers negatively influences the subjects. How long this effect lasts could not be concluded from our survey, as these points were only asked directly before and after the seminar. However, these results might have influenced the learning gain in the video group negatively [33]. A scoping review of transitions in general practice postgraduate training concluded, that challenges in training can “either advance development and contribute positively to professional identity formation and clinical competency, or detract from learning and potentially contribute to burnout and attrition from training programmes” [45]. Hence, it is not clear whether and how feeling less rested and pleasant interacts with long-term learning outcomes or participants’ willingness to perform home visits in the future.

In the VR group changes in the STAI can be interpreted as positive results. This means that negative feelings did not result from immersion in VR or they were adequately addressed in the course of the seminar. Hence, the possibility of triggering negative emotions needs to be considered and potentially discussed by the lecturer. Future studies should address previous traumata in relation to the performance of home visits.

Immediate learning effects using VR and video did not seem to differ. However, the use of VR applications with HMD takes more time and is challenging in terms of logistics. Thus, in a 90-minute seminar, there is a possible risk running out of time to cover the rest of the content. Moreover, we had a loss of participants who did not want to use the technology because they were afraid of the motion sickness symptoms they had experienced in prior confrontations with VR. The other participants showed no sickness from immersion. Visually Induced Motion Sickness (VIMS) is a relevant side effect of using VR technology [46] that needs special consideration, e.g. a back-up plan for those who do not want to use this technology in the seminar. In this study we could offer those participants the video home visits without any hesitations from side of the participants.

Computer-generated VR instead of 360° VR could have been an alternative for creating home visit scenarios with the aim of reducing VIMS introduced by poor image resolutions and alterations at the edges of the pictures that were artificially put together [47]. These approaches need to be used deliberately due to financial efforts. However, VR in the home visit setting can be considered as a supportive digital learning method. Scepticism can be reduced with more experience [48]. It turns out that when VR becomes more commonplace in medical education, it is perceived as positive [49]. This is supported here by the majority of the VR group stating that they would like to use VR in continuing medical education. Those who did not try VR in the video group were more reserved in the assessment of this statement.

What is striking in the socio-demographic distribution of the participants is the wide age variance, which is due to the low-threshold and popular peculiarity in Germany that a change to general practice is easily possible even after years in another speciality [50].

The limitations of our study include the small overall number of participants, particularly in the VR group, which was not sufficient for further statistical analysis. Because of that we were not able to calculate and describe more than descriptive data. Hence, a statistical comparison of the video and VR group was not possible. At the same time, we consider the results from this small study group as a valuable contribution to research to be further built on (51, 52). In addition, the participants were not randomised to VR or video group, as the seminar groups already existed according to the training day in which our seminar was embedded. Confounders, such as previous home visit experiences and experiences with VR technology were assessed, but not included into the analysis of the learning gain, which might lead to biases in the presented results. Especially group differences in home visit experience and age of the participants need to be considered while interpreting the data. However, the seminars were held by the same lecturers with the same content throughout the study in order to reduce further confounding.

Numerous studies have demonstrated the effectiveness of the original STAI and its validated German version, as well as an additional validated 5-item short version that clearly overlaps with the version we used, in describing emotions within VR environments (53–55). However, the 10-item short version of the STAI in German that we use has not yet been validated, which has implications for the reliability and validity of the data collection instrument.

Furthermore, our results rely on the self-assessment of postgraduate trainees, which might not correlate well with objective external measurement, leading to inaccuracies and a lack of comparability. In addition, it is conceivable that over- or underestimation of the participants cannot be ruled out, that a memory bias may have occurred, especially at t2, or that the answers may be distorted by individual expectations of themselves or their professional role. Other forms of evaluation need to be used in order to confirm findings. However, there is evidence in the medical training field, for example, with medical students in Germany, telling the CSA a valid tool to do so because it is suitable for assessing the quality of learning, as it may be a valid advantage in relation to learning objectives [56, 57].

While interaction was made possible in a small group exchange in the debriefing part of the seminar, participants with head-mounted displays in the VR group could not engage with other participants as participants in the video group were able to do in sharing a tablet. Shared displays were found to “establish a social workspace for learning with handheld devices” [58]. Therefore, one might argue that the study design might have been more equivalent in its interventions when each participant would have used a tablet.

As there were only two survey points after the intervention — immediately afterwards and two months later — our work can only provide an indication of lasting effects, not an outlook on long-term results. In general, further research will be needed as our project was planned as pilot study. Small and large effect sizes that were calculated in this study can be used to adequate power calculation.

## Conclusions

Due to the high numbers of violence experienced during home visits, it is essential to address personal safety during home visits in the further training of postgraduate trainees in general practice. To make these potential dangers more understandable, to sharpen the eye for them and learn how to react to them, the use of digital learning can be helpful. There was a well-founded indication in our study that this should be investigated further in future research.

The assessment of our seminar with video and VR scenarios achieved promising results in the short-term results and suggests a positive long-term outlook for learning gains.

The use of the 360° VR application did not result in promising differences. Instead, there are challenges in implementing it as a teaching method that need to be considered.

## Supplementary Information

Supplementary Material 1

## Data Availability

The datasets used and/or analysed during the current study are available from the corresponding author on reasonable request.
