# Supplementary material for: Virtual home visits training regarding personal safety for postgraduate trainees in general practice – learning gain and feelings of anxiety with video and 360° VR applications
Source: BMC Med Educ. 2025 Oct 2;25:1276. doi: 10.1186/s12909-025-07995-x (PMC12490146; doi:10.1186/s12909-025-07995-x)
Supplement: Supplementary file 1 — Supplementary Material 1 [file 12909_2025_7995_MOESM1_ESM.pdf]

# Questionnaire t0 – right at the beginning of the seminar

| **1) QUESTIONS FOR SELF-ASSESSMENT (CSA)**<br><br>**Please mark your self-assessment of the following statements on a scale from "not at all" to "very much".** | | | | | | |
|---|---|---|---|---|---|---|
| | **not at all** | | | | | **very much** |
| **a)** I feel confident to recognise situations jeopardizing the safety of my patients in their home. | ❑ | ❑ | ❑ | ❑ | ❑ | ❑ |
| **b)** I feel confident to be able to make recommendations on situations jeopardizing the safety of my patients in their home. | ❑ | ❑ | ❑ | ❑ | ❑ | ❑ |
| **c)** I feel confident to identify dangerous situations for myself during a home visit. | ❑ | ❑ | ❑ | ❑ | ❑ | ❑ |
| **d)** I feel confident to be able to respond adequately to dangerous situations for myself during a home visit. | ❑ | ❑ | ❑ | ❑ | ❑ | ❑ |
| **e)** I feel confident to recognise the “witnessed” patient’s history. | ❑ | ❑ | ❑ | ❑ | ❑ | ❑ |
| **f)** I feel medically well prepared for performing home visits. | ❑ | ❑ | ❑ | ❑ | ❑ | ❑ |
| **g)** I feel socially well prepared for home visiting. | ❑ | ❑ | ❑ | ❑ | ❑ | ❑ |
| **h)** I feel confident to perform home visits. | ❑ | ❑ | ❑ | ❑ | ❑ | ❑ |
| **i)** I am scared of performing home visits on my own. | ❑ | ❑ | ❑ | ❑ | ❑ | ❑ |

| **2) What experience do you have with home visits? I have...**<br>***(multiple answers possible)*** | |
|---|---|
| ...already performed home visits on my own. | ❑ |
| ...experienced home visits as a companion. | ❑ |
| ...dealt with home visits theoretically. | ❑ |
| ...never got to know home visits. | ❑ |

| **3) How often have you used virtual reality applications so far?** | | | | |
|---|---|---|---|---|
| never used | | | | regularly using |
| ❑ | ❑ | ❑ | ❑ | ❑ |

**4) QUESTIONS ABOUT EXPERIENCED EMOTIONS (STAI)**

**To what extent do the following descriptions of feelings apply to you at the moment? Check the box that applies to you. There are no right or wrong answers. Please don't think about it for long and then decide how strongly you feel the emotion in question at the moment.**

| **The following statements apply to me ...** | **not at all** | | | | | **very much so** |
|---|---|---|---|---|---|---|
| **a)** I feel calm. | ❑ | ❑ | ❑ | ❑ | ❑ | ❑ | ❑ |
| **b)** I am tense. | ❑ | ❑ | ❑ | ❑ | ❑ | ❑ | ❑ |
| **c)** I feel upset. | ❑ | ❑ | ❑ | ❑ | ❑ | ❑ | ❑ |
| **d)** I feel rested. | ❑ | ❑ | ❑ | ❑ | ❑ | ❑ | ❑ |
| **e)** I feel anxious. | ❑ | ❑ | ❑ | ❑ | ❑ | ❑ | ❑ |
| **f)** I feel self-confident. | ❑ | ❑ | ❑ | ❑ | ❑ | ❑ | ❑ |
| **g)** I feel nervous. | ❑ | ❑ | ❑ | ❑ | ❑ | ❑ | ❑ |

| 4) QUESTIONS ABOUT EXPERIENCED EMOTIONS (STAI)<br>To what extent do the following descriptions of feelings apply to you at the moment? Check the box that applies to you. There are no right or wrong answers. Please don't think about it for long and then decide how strongly you feel the emotion in question at the moment. | | | | | | | |
|---|---|---|---|---|---|---|---|
| **The following statements apply to me …** | **not at all** | | | | | | **very much so** |
| **h)** I feel „high-strung”. | ❑ | ❑ | ❑ | ❑ | ❑ | ❑ | ❑ |
| **i)** I am worried. | ❑ | ❑ | ❑ | ❑ | ❑ | ❑ | ❑ |
| **j)** I feel pleasant. | ❑ | ❑ | ❑ | ❑ | ❑ | ❑ | ❑ |

| 5) How empathetic do you think you are? | | | | |
|---|---|---|---|---|
| **not at all** | | | | **very much** |
| ❑ | ❑ | ❑ | ❑ | ❑ |

| 6) How well can you tolerate diagnostic uncertainty? | | | | | |
|---|---|---|---|---|---|
| **not at all** | | | | **very much** | **I do not know** |
| ❑ | ❑ | ❑ | ❑ | ❑ | ❑ |

| 7) How important is he „witnessed” patient’s history to you? | | | | | |
|---|---|---|---|---|---|
| **not at all** | | | | **very much** | **I do not know** |
| ❑ | ❑ | ❑ | ❑ | ❑ | ❑ |

| 8) How important is your patients' continuity of care to you? | | | | | |
|---|---|---|---|---|---|
| not at all | | | | very much | I do not know |
| ❑ | ❑ | ❑ | ❑ | ❑ | ❑ |

| 9a) Which gender do you feel you belong to? | ❑ female | | ❑ male | | ❑ diverse |
|---|---|---|---|---|---|
| b) In which year were you born? | | | | | |
| c) What year of specialty training in general practice are you in? | 1 ❑ | 2 ❑ | 3 ❑ | 4 ❑ | 5 ❑ |
| d) Do you have any previous medical training? | no ❑ | | | yes ❑ | |
| If yes, which one? | | | | | |

| 10) From your perspective, what other aspects of the topic are important that we have not asked about? |
|---|
| |

# Questionnaire t1 – right after the seminar

| **1) QUESTIONS FOR SELF-ASSESSMENT (CSA)**<br>**Please mark your self-assessment of the following statements on a scale from "not at all" to "very much".** | | | | | | |
|---|---|---|---|---|---|---|
| | **not at all** | | | | | **very much** |
| **a)** I feel confident to recognise situations jeopardizing the safety of my patients in their home. | ❑ | ❑ | ❑ | ❑ | ❑ | ❑ |
| **b)** I feel confident to be able to make recommendations on situations jeopardizing the safety of my patients in their home. | ❑ | ❑ | ❑ | ❑ | ❑ | ❑ |
| **c)** I feel confident to identify dangerous situations for myself during a home visit. | ❑ | ❑ | ❑ | ❑ | ❑ | ❑ |
| **d)** I feel confident to be able to respond adequately to dangerous situations for myself during a home visit. | ❑ | ❑ | ❑ | ❑ | ❑ | ❑ |
| **e)** I feel confident to recognise the “witnessed” patient’s history. | ❑ | ❑ | ❑ | ❑ | ❑ | ❑ |
| **f)** I feel confident to perform home visits. | ❑ | ❑ | ❑ | ❑ | ❑ | ❑ |
| **g)** I am scared of performing home visits on my own. | ❑ | ❑ | ❑ | ❑ | ❑ | ❑ |

| **2) QUESTIONS ABOUT EXPERIENCED EMOTIONS (STAI)**<br>**To what extent do the following descriptions of feelings apply to you at the moment? Check the box that applies to you. There are no right or wrong answers. Please don't think about it for long and then decide how strongly you feel the emotion in question at the moment.** | | | | | | | |
|---|---|---|---|---|---|---|---|
| **The following statements apply to me …** | **not at all** | | | | | | **very much so** |
| **a)** I feel calm. | ❑ | ❑ | ❑ | ❑ | ❑ | ❑ | ❑ |

| 2) QUESTIONS ABOUT EXPERIENCED EMOTIONS (STAI)<br>To what extent do the following descriptions of feelings apply to you at the moment? Check the box that applies to you. There are no right or wrong answers. Please don't think about it for long and then decide how strongly you feel the emotion in question at the moment. | | | | | | | |
|---|---|---|---|---|---|---|---|
| **The following statements apply to me …** | **not at all** | | | | | | **very much so** |
| **b)** I am tense. | ❑ | ❑ | ❑ | ❑ | ❑ | ❑ | ❑ |
| **c)** I feel upset. | ❑ | ❑ | ❑ | ❑ | ❑ | ❑ | ❑ |
| **d)** I feel rested. | ❑ | ❑ | ❑ | ❑ | ❑ | ❑ | ❑ |
| **e)** I feel anxious. | ❑ | ❑ | ❑ | ❑ | ❑ | ❑ | ❑ |
| **f)** I feel self-confident. | ❑ | ❑ | ❑ | ❑ | ❑ | ❑ | ❑ |
| **g)** I feel nervous. | ❑ | ❑ | ❑ | ❑ | ❑ | ❑ | ❑ |
| **h)** I feel „high-strung". | ❑ | ❑ | ❑ | ❑ | ❑ | ❑ | ❑ |
| **i)** I am worried. | ❑ | ❑ | ❑ | ❑ | ❑ | ❑ | ❑ |
| **j)** I feel pleasant. | ❑ | ❑ | ❑ | ❑ | ❑ | ❑ | ❑ |

**3) From your perspective, what other aspects of the topic are important that we have not asked about?**

# Questionnaire t2 – two months later

| **1) QUESTIONS FOR SELF-ASSESSMENT (CSA)**<br>**Please mark your self-assessment of the following statements on a scale from "not at all" to "very much".** | | | | | | |
|---|---|---|---|---|---|---|
| | **not at all** | | | | | **very much** |
| **a)** I feel confident to recognise situations jeopardizing the safety of my patients in their home. | ❑ | ❑ | ❑ | ❑ | ❑ | ❑ |
| **b)** I feel confident to be able to make recommendations on situations jeopardizing the safety of my patients in their home. | ❑ | ❑ | ❑ | ❑ | ❑ | ❑ |
| **c)** I feel confident to identify dangerous situations for myself during a home visit. | ❑ | ❑ | ❑ | ❑ | ❑ | ❑ |
| **d)** I feel confident to be able to respond adequately to dangerous situations for myself during a home visit. | ❑ | ❑ | ❑ | ❑ | ❑ | ❑ |
| **e)** I feel confident to recognise the "witnessed" patient's history. | ❑ | ❑ | ❑ | ❑ | ❑ | ❑ |
| **f)** I feel medically well prepared for performing home visits. | ❑ | ❑ | ❑ | ❑ | ❑ | ❑ |
| **g)** I feel socially well prepared for home visiting. | ❑ | ❑ | ❑ | ❑ | ❑ | ❑ |
| **h)** I feel confident to perform home visits. | ❑ | ❑ | ❑ | ❑ | ❑ | ❑ |
| **i)** I am scared of performing home visits on my own. | ❑ | ❑ | ❑ | ❑ | ❑ | ❑ |

| **2) What experience do you have with home visits? I have...**<br>***(multiple answers possible)*** | |
|---|---|
| …already performed home visits on my own. | ❑ |
| …experienced home visits as a companion. | ❑ |

| | |
|---|---|
| …dealt with home visits theoretically. | ❑ |
| …never got to know home visits. | ❑ |

| **3) EXPERIENCE WITH VIRTUAL REALITY**<br>**Please use the following scale to rate the extent to which you agree with the statement.** | | | | | |
|---|---|---|---|---|---|
| | **not at all** | | | | **very much** |
| I think I would like to use the VR software a lot. | ❑ | ❑ | ❑ | ❑ | ❑ |

| **4) How empathetic do you think you are?** | | | | |
|---|---|---|---|---|
| not at all | | | | very much |
| ❑ | ❑ | ❑ | ❑ | ❑ |

| **5) How well can you tolerate diagnostic uncertainty?** | | | | | |
|---|---|---|---|---|---|
| not at all | | | | very much | I do not know |
| ❑ | ❑ | ❑ | ❑ | ❑ | ❑ |

| **6) How important is the "witnessed" patient's history to you?** | | | | | |
|---|---|---|---|---|---|
| not at all | | | | very much | I do not know |
| ❑ | ❑ | ❑ | ❑ | ❑ | ❑ |

| **7) How important is your patients' continuity of care to you?** | | | | | |
|---|---|---|---|---|---|
| not at all | | | | very much | I do not know |
| ❑ | ❑ | ❑ | ❑ | ❑ | ❑ |

**8) From your perspective, what other aspects of the topic are important that we have not asked about?**
